# Poll Finds Rural Residents More Hesitant to Get Vaccinated

**DOI:** 10.13023/jah.0301.07

**Published:** 2021-01-24

**Authors:** Tim Marema

**Keywords:** Appalachia, COVID-19, vaccine, rural, rates of infection

## Abstract

Rural residents are more hesitant than their metropolitan counterparts to get a Covid-19 vaccination, even though rural areas have higher rates of infections and deaths from the coronavirus.

Rural residents are more hesitant than their metropolitan counterparts to get a COVID-19 vaccination, even though rural areas have higher rates of infections and deaths from the coronavirus, according to a new report.

**Figure:**
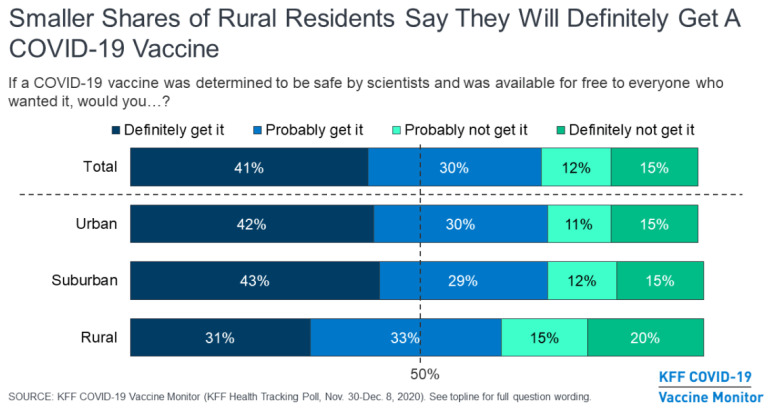


About a third (35%) of people living in rural areas said they probably would not or definitely would not get a COVID-19 vaccine, compared to about a quarter of suburban (27%) and urban residents (26%) who said the same.

The increased reluctance of rural residents to get vaccinated for COVID-19 was evident even when researchers controlled for other factors such as age, education, and party affiliation.

The poll, part of the Kaiser Family Foundation’s vaccine monitor project was conducted November 30 to December 8, the week before the first doses of COVID-19 were administered in the U.S.

The poll asked approximately 1,700 respondents whether they would get a vaccine if it was free, safe, and effective.

Party affiliation was the biggest indicator of whether a person said they would refuse vaccination. Forty-two percent of Republicans said they probably or definitely would not get vaccinated. Only 12% of Democrats said they would not take the vaccine.

**Figure:**
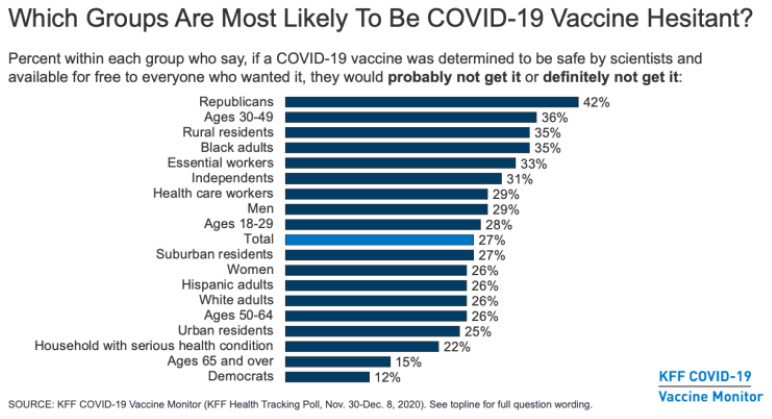


Rural respondents gave three main reasons for refusing vaccination:

- They were not worried that they or a family member will get sick from the coronavirus.
- The seriousness of the pandemic is overblown.
- And vaccination is more of a personal choice than a community responsibility.

**Figure:**
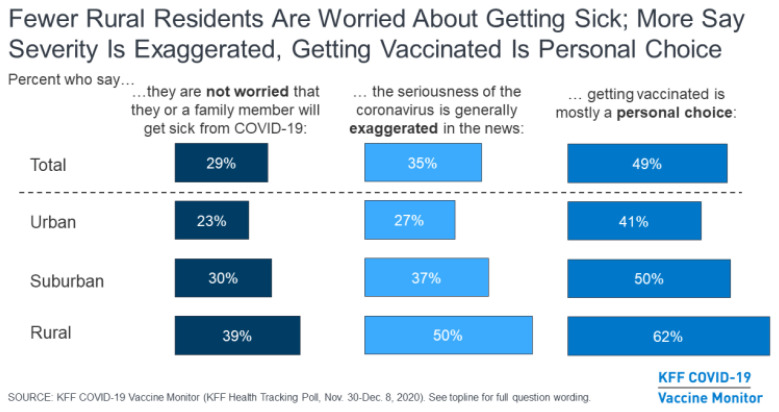


Last month, rural residents were twice as likely to die from COVID-19 than residents of the nation’s largest cities, according to Daily Yonder analysis. In December, there were 35.1 deaths per 100,000 from COVID-19–related causes in rural areas, versus 17.7 deaths per 100,000 in metropolitan areas with 1 million or more residents. The rural death rate has been higher than the metropolitan rate since mid-August.

Attitudes about vaccination are not fixed, however. The survey found that feelings about vaccination improved since September, when a similar poll was conducted. In September, 63% of respondents nationally said they would probably or definitely get vaccinated. By December, that number had risen to 71%.

The survey used randomly generated landline and cellphone numbers to select respondents. Participants in previous polls were also part of the survey. The data was weighted to balance the survey sample with key national population characteristics such as sex, age, education, race, region, and other factors.

Rural respondents were defined as residents of counties that lie outside metropolitan statistical areas, using the Office of Management and Budget county categorization system.

More information about the poll is available in a topline and methodology report. See Additional Files.

## Supplementary Information



