# Supplementary material for: Poll Finds Rural Residents More Hesitant to Get Vaccinated
Source: J Appalach Health. 2021 Jan 24;3(1):61–5. doi: 10.13023/jah.0301.07 (PMC9138735; doi:10.13023/jah.0301.07)
Supplement: Supplementary file 1 [file 3.1.7Maremaadditionalfile.pdf]

Topline

KFF Health Tracking Poll/ KFF COVID-19 Vaccine Monitor

December 2020

METHODOLOGY

This *KFF Health Tracking Poll/ KFF COVID-19 Vaccine Monitor* was designed and analyzed by public opinion researchers at the Kaiser Family Foundation (KFF). The survey was conducted November 30- December 8, 2020, among a nationally representative random digit dial telephone sample of 1,676 adults ages 18 and older (including interviews from 298 Hispanic adults and 390 non-Hispanic Black adults), living in the United States, including Alaska and Hawaii (note: persons without a telephone could not be included in the random selection process). Phone numbers used for this study were randomly generated from cell phone and landline sampling frames, with an overlapping frame design, and disproportionate stratification aimed at reaching Hispanic and non-Hispanic Black respondents. The sample also includes interviews completed with respondents who had previously completed an interview on the KFF Tracking Poll (*n* =267) or an interview on the SSRS Omnibus poll (and other RDD polls) and identified as Hispanic (*n* = 80; including 14 in Spanish) or non-Hispanic Black (*n*=179). Computer-assisted telephone interviews conducted by landline (391) and cell phone (1,285, including 947 who had no landline telephone) were carried out in English and Spanish by SSRS of Glen Mills, PA. To efficiently obtain a sample of lower-income and non-White respondents, the sample also included an oversample of prepaid (pay-as-you-go) telephone numbers (25% of the cell phone sample consisted of prepaid numbers) Both the random digit dial landline and cell phone samples were provided by Marketing Systems Group (MSG). For the landline sample, respondents were selected by asking for the youngest adult male or female currently at home based on a random rotation. If no one of that gender was available, interviewers asked to speak with the youngest adult of the opposite gender. For the cell phone sample, interviews were conducted with the adult who answered the phone. KFF paid for all costs associated with the survey.

The combined landline and cell phone sample was weighted to balance the sample demographics to match estimates for the national population using data from the Census Bureau's 2019 U.S. American Community Survey (ACS), on sex, age, education, race, Hispanic origin, and region, within race-groups, along with data from the 2010 Census on population density. The sample was also weighted to match current patterns of telephone use using data from the January- June 2019 National Health Interview Survey. The weight takes into account the fact that respondents with both a landline and cell phone have a higher probability of selection in the combined sample and also adjusts for the household size for the landline sample, and design modifications, namely, the oversampling of prepaid cell phones and likelihood of non-response for the re-contacted sample. All statistical tests of significance account for the effect of weighting.

The margin of sampling error including the design effect for the full sample is plus or minus 3 percentage points. Numbers of respondents and margins of sampling error for key subgroups are shown in the table below. For results based on other subgroups, the margin of sampling error may be higher. Sample sizes and margins of sampling error for other subgroups are available by request. Note that sampling error is only one of many potential sources of error in this or any other public opinion poll. Kaiser Family Foundation public opinion and survey research is a charter member of the Transparency Initiative of the American Association for Public Opinion Research.

Group	N (unweighted)	M.O.S.E.
Total	1,676	± 3 percentage points
**Race/Ethnicity**		
White, non-Hispanic	842	± 4 percentage points
Black, non-Hispanic	390	± 7 percentage points
Hispanic	298	± 7 percentage points
**Party Identification**		
Democrat	617	± 5 percentage points
Republican	382	± 6 percentage points
Independent	478	± 5 percentage points
**Vaccine Uptake**		
Definitely/probably would get vaccine	1,213	± 4 percentage points
Definitely/probably would not get vaccine	427	± 6 percentage points

Notes for reading the topline:
- Percentages may not always add up to 100 percent due to rounding.
- Values less than 0.5 percent are indicated by an asterisk (*).
- "Vol." indicates a response was volunteered by the respondent, not offered as an explicit choice
- Questions are presented in the order asked; question numbers may not be sequential.

All trends shown in this document come from the KFF Health Tracking Polls except for:
09/20: KFF/The Undefeated Poll on Race and Health (August 20-September 14, 2020)
07/20: KFF Poll: Views of Doctors, Nurses, Insurance Companies, and Drug Companies (July 14-19, 2020)

HISPANIC. Are you, yourself, of Hispanic or Latino background, such as Mexican, Puerto Rican, Cuban, or some other Spanish background?

	12/20
Yes	16
No	83
Don't know	*
Refused	1

RACE. What is your race? Are you white, black, Asian or some other race? (IF RESPONDENT SAYS HISPANIC ASK: Do you consider yourself a white Hispanic or a black Hispanic? CODE AS WHITE (1) OR BLACK (2). IF RESPONDENTS REFUSED TO PICK WHITE OR BLACK HISPANIC, RECORD HISPANIC AS "OTHER," CODE 97)

Race/Hispanic Combo Table

Based on total

	12/20
White, non-Hispanic	61
Total non-White	39
Black or African-American, non-Hispanic	12
Hispanic	16
Asian, non-Hispanic	3
Other/Mixed race, non-Hispanic	6
Undesignated	3

NATIVITY. Were you born in the United States (IF HISPANIC=1 INSERT, on the island of Puerto Rico), or in another country?

Based on Hispanics

	12/20
U.S.	49
Puerto Rico	5
Another country	47
Don't know	-
Refused	*
	n=298

ACA. As you may know a health reform bill was signed into law in 2010, known commonly as the Affordable Care Act or Obamacare. Given what you know about the health reform law, do you have a generally (favorable) or generally (unfavorable) opinion of it? [GET ANSWER THEN ASK: Is that a very (favorable/unfavorable) or somewhat (favorable/unfavorable) opinion?]

	Very favorable	Somewhat favorable	Somewhat unfavorable	Very unfavorable	Don't know/ Refused
12/20	32	21	11	23	13
10/20	29	26	14	26	6
9/20	26	23	14	28	9
7/20	31	20	11	25	13
5/20	24	27	14	27	8
Early 4/20	22	28	17	22	12
02/20	24	31	14	23	9
01/20	28	25	11	26	10
11/19	26	26	12	29	7
10/19	30	21	12	28	8
09/19	29	24	15	26	7
07/19	26	22	12	29	12
06/19	27	19	13	27	12
04/19	29	21	11	27	13
03/19	27	23	11	28	11
02/19	28	22	12	25	12
01/19	29	22	12	28	10
11/18	28	25	14	26	8
09/18	23	26	15	27	9
08/18	26	24	13	27	10
07/18	28	20	12	28	11
06/18	26	24	13	28	8
04/18	27	22	15	28	9
03/18	26	24	15	28	7
02/18	33	21	12	30	5
01/18	27	23	12	30	8
11/17	29	21	17	29	5
10/17	29	22	13	27	9
09/17	27	19	14	30	10
08/17	30	22	10	29	8
07/17	28	22	12	32	6
06/17	29	22	14	27	8
05/17	29	20	13	29	9
Late 04/17[1]	24	24	15	26	12
Early 04/17	20	26	16	30	9
03/17	21	28	15	29	6
02/17	20	28	15	27	10
12/16	21	22	16	30	11
11/16	19	24	17	28	11
10/16	19	26	13	32	10
09/16	18	26	17	30	9
08/16	19	21	14	28	17
07/16	18	22	17	29	14
06/16	18	24	12	32	14
04/16	15	23	17	32	13
03/16	21	20	17	30	13
02/16	19	22	12	34	14
01/16	21	20	16	28	16
12/15	19	21	13	33	14
11/15	19	19	17	28	17
10/15	21	21	15	27	16
09/15	21	20	15	30	14
08/15	23	21	16	25	14
06/29/15	23	20	13	27	17

[1] January 2012 through Late April 2017 trend wording was "As you may know, a health reform bill was signed into law in 2010..."

ACA continued…

	Very favorable	Somewhat favorable	Somewhat unfavorable	Very unfavorable	Don't know/ Refused
06/09/15	19	20	16	26	19
04/15	22	21	15	27	14
03/15	22	19	15	28	16
01/15	19	21	16	30	15
12/14	18	23	16	30	14
11/14	18	19	16	30	18
10/14	16	20	16	27	20
09/14	15	20	15	32	19
07/14	15	22	18	35	11
06/14	19	20	15	30	16
05/14	19	19	12	33	17
04/14	19	19	16	30	16
03/14	18	20	14	32	15
02/14	16	19	14	33	18
01/14	17	17	15	35	16
12/13	17	17	12	36	18
11/13	15	18	13	36	18
10/13	21	17	13	31	18
09/13	20	19	13	30	17
08/13	17	20	14	28	20
06/13	15	20	13	30	23
04/13	16	19	12	28	24
03/13	17	20	13	27	23
02/13	18	18	13	29	23
11/12	19	24	12	27	19
10/12	20	18	14	29	19
09/12	25	20	12	28	14
08/12	21	17	13	30	19
07/12	20	18	13	31	17
06/12	25	16	11	30	18
05/12	17	20	12	32	19
04/12	20	22	9	34	15
03/12	18	23	11	29	19
02/12	17	25	16	27	15
01/12	18	19	14	30	19
12/11[2]	19	22	15	28	17
11/11	17	20	15	29	19
10/11	12	22	20	31	15
09/11	18	23	14	29	16
08/11	16	23	17	27	17
07/11	20	22	12	31	15
06/11	15	27	16	30	12
05/11	19	23	15	29	14
04/11	20	21	14	27	18
03/11	21	21	15	31	13
02/11	16	27	19	29	8
01/11	19	22	16	34	9
12/10[3]	22	20	14	27	18
11/10	19	23	12	28	18
10/10	18	24	15	29	15
09/10	19	30	15	25	11
08/10	19	24	13	32	12
07/10	21	29	10	25	14

[2] February 2011 through December 2011 trend wording was "As you may know, a health reform bill was signed into law early last year. Given what you know about the health reform law, do you have a generally (favorable) or generally (unfavorable) opinion of it? (Is that a very favorable/unfavorable or somewhat favorable/unfavorable opinion?)"

[3] May 2010 through December 2011 trend wording was "As you may know, a health reform bill was signed into law earlier this year…"

ACA continued...

	Very favorable	Somewhat favorable	Somewhat unfavorable	Very unfavorable	Don't know/ Refused
06/10	20	28	16	25	10
05/10	14	27	12	32	14
04/10[4]	23	23	10	30	14

Q1. What would you like to see the next presidential administration and Congress do when it comes to the health care law? (rotate 1-4/4-1)

	Build on what the law does	Keep the law as it is	Scale back what the law does	Repeal the entire law	None of these/ Something else(Vol.)	Don't know	Refused[5]
12/20	48	14	9	20	2	7	1
11/16[6]	30	19	17	26	3	3	1
10/16[7]	31	18	9	32	6	3	1
06/16[8]	28	17	11	33	5	7	--
04/16	30	14	11	32	6	7	--
01/16	30	20	12	30	2	5	--
12/15	22	18	14	35	4	7	--
11/15	26	16	12	30	6	10	--
10/15	28	16	11	32	5	7	--
09/15	25	18	11	31	5	9	--
08/15	28	22	12	28	4	5	--
06/29/15	25	22	12	27	5	7	--
06/09/15	24	19	12	29	7	10	--
04/15	24	22	12	29	5	8	--
03/15	23	23	10	30	7	7	--
01/15	23	19	14	32	5	7	--
12/14	24	21	12	31	4	7	--
11/14	22	20	17	29	5	8	--

4 April 2010 trend wording was "President Obama did sign a health reform bill into law last month…Given what you know about the new health reform law, do you have a generally (favorable) or generally (unfavorable) opinion of it? (Is that a very favorable/unfavorable or somewhat favorable/unfavorable opinion?)"

5 Don't know and Refused were recorded separately for this question and some other questions throughout the survey. Prior to October 2016, Don't know/Refused was combined into one category. Trend results prior to 2016 for "Refused" are shown in the "Don't know" category.

6 November 2016 question wording was "What would you like to see President-elect Donald Trump and the next Congress do when it comes to the health care law? Expand what the law does, move forward with implementing the law as it is, scale back what the law does, or repeal the entire law?"

7 October 2016 question wording was "What would you like to see the next president and Congress do when it comes to the health care law?"

8 November 2014 to April 2016 question wording was "What would you like to see Congress do when it comes to the health care law?"

Q2. I'm going to read you some different health care proposals. For each one, please say whether you (favor) or (oppose) this proposal. First (ITEM). Do you (favor) or (oppose) this proposal? What about (NEXT ITEM)? IF NECESSARY: Do you (favor) or (oppose) this proposal? (rotate text in parentheses; scramble a-g)

	Favor	Oppose	Don't know/ Refused	
a. Lowering the age when people become eligible for Medicare from 65 to 60				
12/20	65	30	4	*n=1,676*
b. Expanding government financial help for those who buy their own insurance on the marketplace				
12/20	66	27	7	*n=1,676*
c. Protecting patients from surprise medical bills by prohibiting health care providers from charging high out-of-network prices when patients are hospitalized or need emergency care				
12/20	80	18	2	*n=1,676*
d. Having a government-administered health plan, sometimes called a public option, that would compete with private health insurance plans and be available as an option to all Americans				
12/20	71	26	4	*n=1,676*
e. Allowing the federal government to negotiate with drug companies to get a lower price on medications that would apply to both Medicare and private insurance				
12/20	89	10	1	*n=1,676*
10/19	85	13	2	*n=1,205*
f. Making information about the price of doctors' visits, tests, and procedures more available to patients				
12/20	93	6	1	*n=1,676*
g. Guaranteeing health insurance coverage to lower-income people whose states have not expanded their Medicaid program				
12/20	76	20	5	*n=1,676*

Q3. Do you feel that worry or stress related to coronavirus has had a negative impact on your mental health, or not? (IF YES ASK: Was that a major impact or a minor impact?)

	12/20	7/20	5/20	Early 4/20	3/20
Yes (Net)	51	53	39	45	32
Yes – major impact	25	26	12	19	14
Yes – minor impact	26	28	27	26	18
No	48	45	60	54	67
I don't have worry or stress related to coronavirus (Vol.)	*	1	*	*	-
Don't know/Refused (NET)	1	1	*	1	1
Don't know	1	1	*	1	1
Refused	*	-	-	-	*
	n=1,676	*n=1,313*	*n=1,189*	*n=1,226*	*n=1,216*

Q27. Starting today, how much longer do you think you can follow social distancing in order to limit the spread of coronavirus in your community?[9] Can you not do it at all, or can you do it for less than a month, between 1 and 3 months, between 4 and 6 months, or more than 6 months or until there is a vaccine widely available?

	12/20
Not do it at all	9
Less than a month/1-3 months (NET)	12
Less than a month	4
Between 1 and 3 months	9
4-6 months/More than 6 months (NET)	75
Between 4 and 6 months	5
More than 6 months or until there is a vaccine widely available	70
Can follow longer, but won't because need freedom (Vol.)	1
Don't know/Refused (NET)	3
Don't know	2
Refused	1
	n=1,676

Q4. Which of the following best describes your feelings about the coronavirus outbreak in the United States? (rotate response options 1-2/2-1, keep Option A and Option B text from rotating)

	12/20	10/20	9/20	7/20	5/20	Late 4/20	Early 4/20
Option A: The worst is behind us	25	33	38	20	28	31	13
Option B: The worst is yet to come	51	42	38	60	50	51	74
The coronavirus is or will not be a major problem in the U.S.	19	20	19	16	16	13	10
Don't know	4	4	4	4	6	4	2
Refused	1	1	*	1	1	1	*
	n=1,676	*n=1,207*	*n=1,199*	*n=1,313*	*n=1,189*	*n=1,202*	*n=1,226*

Q5. Thinking about what is said in the news, in your view is the seriousness of coronavirus (generally exaggerated), generally correct, or is it (generally underestimated)? (rotate text in parentheses)

	12/20
Generally exaggerated	35
Generally correct	36
Generally underestimated	25
Don't know	3
Refused	1

Q6. How worried, if at all, are you that you or someone in your family will get sick from the coronavirus ? Are you very worried, somewhat worried, not too worried, or not at all worried?

	Very/ Somewhat worried (NET)	Very worried	Somewhat worried	Not too/Not at all worried (NET)	Not too worried	Not at all worried	Not Applicable (Vol)	Don't Know/ Refused (NET)	
12/20	68	30	38	29	15	14	3	*	*n=1,676*
10/20	66	35	31	31	18	12	3	*	*n=1,207*
Early 4/20	53	28	24	41	17	24	6	*	*n=1,226*
3/20	62	27	36	37	21	16	-	*	*n=1,216*

[9] Late 4/20 trend wording was "Starting today, how much longer do you think you can follow strict social distancing and sheltering in place guidelines in order to limit the spread of coronavirus in your community?"

2/20[10]	43	22	21	56	33	23	-	1	*n=1,207*

Q7. In its efforts to slow the spread of coronavirus, do you think your state currently has (too many), (not enough), or about the right amount of restrictions on (INSERT ITEM)? (rotate text in parentheses; scramble a-b)

	Too many	Not enough	About the right time	Don't know	Refused
a. Businesses					
12/20	25	32	40	2	*
b. Individuals					
12/20	20	36	42	2	*

Q8. How often, if at all, do you wear a protective mask when you leave your house and might be in contact with other people? [READ LIST]

	12/20	5/20
Some of the time/Most of the time/Every time(NET)	96	87
At least most of the time (NET)	89	73
Every time	73	52
Most of the time	16	21
Some of the time/Never (NET)	11	26
Some of the time	7	14
Never	4	12
Don't know/Refused (NET)	*	*
Don't know	*	*
Refused	-	*
	n=1,676	*n=1,189*

Q9. Which comes closer to your view: wearing a mask to prevent the spread of COVID-19 (is a personal choice) OR wearing a mask (is part of everyone's responsibility to protect the health of others)? (rotate text in parentheses)

	12/20
Wearing a mask is a personal choice	23
Wearing a mask is part of everyone's responsibility to protect the health of others	73
Both (Vol.)	3
Neither (Vol.)	1
Don't know	*
Refused	*

[10] February 2020 trend wording was "How concerned, if at all, are you that you or someone in your family will get sick from the coronavirus" They or a family member has already gotten sick from coronavirus was volunteered response.

Q10. As far as you know, (INSERT ITEM), or not? (scramble a-b)

	Yes	No	Don't know	Refused	
a. Does wearing a face mask help to limit the spread of coronavirus					
12/20	78	17	5	*	*n=1,676*
10/20	81	17	2	*	*n=1,207*
9/20	81	16	2	*	*n=1,199*
b. Is wearing a face mask harmful to your health					
12/20	21	77	3	*	*n=1,676*
10/20	18	80	2	*	*n=1,207*
9/20	20	77	2	*	*n=1,199*
c. Does wearing a face mask help protect you from coronavirus					
12/20	70	25	5	*	*n=1,676*

Q16. If a coronavirus vaccine was determined to be safe by scientists and was available for free to everyone who wanted it, would you definitely get it, probably get it, probably not get it or definitely not get it?

	12/20	09/20[11]
Would get it (NET)	71	63
Definitely get it	41	34
Probably get it	30	29
Would not get it (NET)	27	34
Probably not get it	12	14
Definitely not get it	15	20
Don't know	2	2
Refused	*	*
Web Blank	-	*
	n=1,676	*n=1,769*

Q17. When a vaccine for COVID-19 is approved by the FDA and widely available to anyone who wants it, do you think you will…? (READ LIST)

Based on those who definitely or probably would get the vaccine or probably not get the vaccine

	12/20
Get the vaccine as soon as you can	41
Wait until it has been available for a while to see how it is working for other people	47
Only get the vaccine if you are required to do so for work, school, or other activities	11
Don't know	1
Refused	1
	n=1,405

Q16/Q17 Combo table

Based on total

	12/20
Definitely get it/Probably get it/Probably not get it (NET)	83
Get the vaccine as soon as you can	34
Wait until it has been available for a while to see how it is working for other people	39
Only get the vaccine if you are required to do so for work, school, or other activities	9
Don't know/Refused	1
Definitely not get it	15
Don't know/Refused	2

[11] Trend from KFF Undefeated Survey on Race and Health, September 2020

Q18. Please tell me if each of the following is a major reason, a minor reason, or not a reason why you would (IF Q16=3 INSERT 'probably') NOT get a COVID-19 vaccine? First (INSERT ITEM), is this is a major reason, a minor reason, or not a reason why you would (IF Q16=3 INSERT 'probably') NOT get a COVID-19 vaccine? What about (INSERT ITEM)? IF NECESSARY: Is this is a major reason, a minor reason, or not a reason why you would (IF Q16=3 INSERT 'probably') NOT get a COVID-19 vaccine? (scramble a-j)

Based on those who definitely or probably would not get the vaccine

	A reason (NET)	Major reason	Minor reason	Not a reason	Don't know/ Refused	
a. You don't trust vaccines in general						
12/20	62	37	25	36	2	*n=427*
b. You don't think you are at risk of getting sick from COVID-19						
12/20	51	20	30	46	4	*n=427*
c. The risks of COVID-19 are being exaggerated						
12/20	66	43	23	31	3	*n=427*
e. The vaccine is too new and you want to wait and see how it works for other people						
12/20	69	53	16	30	1	*n=427*
f. You do not trust the health care system						
12/20	60	35	25	38	2	*n=427*
g. You do not trust the government to make sure the vaccine is safe and effective						
12/20	76	55	21	23	2	*n=427*
h. You are worried about possible side effects						
12/20	81	59	21	18	1	*n=427*
i. You are worried that you may get COVID-19 from the vaccine						
12/20	48	27	21	50	1	*n=427*
j. Politics has played too much of a role in the vaccine development process						
12/20	71	51	19	27	3	*n=427*

Q11. Which comes closer to your view: getting vaccinated against COVID-19 (is a personal choice) OR getting vaccinated (is part of everyone's responsibility to protect the health of others)? (rotate text in parentheses)

	12/20
Getting vaccinated is a personal choice	49
Getting vaccinated is part of everyone's responsibility to protect the health of others	49
Both (Vol.)	2
Neither (Vol.)	*
Don't know	1
Refused	*

Q12. How much do you trust (INSERT ITEM) to provide reliable information about a COVID-19 vaccine? How about (INSERT NEXT ITEM)? IF NECESSARY: Do you trust him/them a great deal, a fair amount, not much or not at all to provide reliable information about a COVID-19 vaccine?

	A great deal/fair amount (NET)	A great deal	A fair amount	Not much/ Not at all (NET)	Not much	Not at all	Depends (Vol.)	Don't know/ Refused
a. President Trump								
12/20	34	19	16	64	16	48	*	2
b. The U.S. Centers for Disease Control and Prevention, or CDC								
12/20	73	32	41	26	16	9	*	1
c. The U.S. Food and Drug Administration, or FDA								
12/20	70	25	45	28	18	10	*	2
d. Dr. Anthony Fauci, the director of the National Institute of Allergy and Infectious Diseases								
12/20	68	39	28	28	13	15	*	4
e. President-elect Joe Biden								
12/20	57	27	30	40	15	26	*	2
f. Your state government officials								
12/20	58	20	38	40	20	20	*	2
g. Your local public health department								
12/20	70	27	42	27	16	11	*	3
h. Your own doctor or health care provider								
12/20	85	51	34	12	7	5	1	2
i. Pharmaceutical companies								
12/20	53	13	40	45	24	21	*	1

9/20 Trend for comparison[12]

	A great deal	A fair amount	Not much	Not at all	Depends (Vol)	Don't know/ Refused (NET)	
a. President Trump							
9/20	21	19	16	43	*	1	*n=1,199*
Early 4/20	21	25	21	32	*	1	*n= 1,226*
3/20	23	23	18	33	*	3	*n= 1,216*
b. The U.S. Centers for Disease Control and Prevention, or CDC							
9/20	25	42	21	10	*	1	*n=1,199*
Early 4/20	43	41	13	3	-	1	*n= 1,226*
3/20	48	37	10	3	*	2	*n= 1,216*
c. Dr. Anthony Fauci, the director of the National Institute of Allergy and Infectious Diseases							
9/20	37	31	15	11	*	6	*n=1,199*
Early 4/20	41	37	10	2	-	10	*n= 609*

[12] September 2020 trend wording was "How much do you trust (INSERT ITEM) to provide reliable information on coronavirus? Do you trust (Him/Them) a great deal, a fair amount, not much or not at all to provide reliable information on coronavirus?)

Q13. Do you think the development and testing of a vaccine for COVID-19 is moving (too quickly), (too slowly), or about right? (rotate text in parentheses)

	12/20
Too quickly	22
Too slowly	12
About right	64
Don't know	2
Refused	*

Q14. Do you think pharmaceutical companies working on a COVID-19 vaccine are (mostly interested in working for the good of the public), (mostly interested in making a profit), or are equally interested in working for the public and making a profit? (rotate text in parentheses)

	12/20
Mostly interested in working for the good of the public	8
Mostly interested in making a profit	32
Equally interested in working for the public and making a profit	58
Don't know	1
Refused	1

07/20 Trend for comparison

Please tell me whether you think drug companies (are mostly interested in working for the good of the public), (are mostly interested in making a profit), or are equally interested in working for the public and making a profit? (rotate text in parentheses)

	07/20
Mostly interested in working for the good of the public	4
Mostly interested in making a profit	76
Equally interested in working for the public and making a profit	19
Don't know	*
Refused	*

Q15. When do you think a vaccine for COVID-19 will be widely available for anyone who wants it in the U.S.? (READ LIST)

	12/20
By the end of 2020	6
Early in 2021	25
By the summer of 2021	40
By the end of 2021	19
Sometime in 2022 or later	8
Don't know	2
Refused	*

ROTATE Q19 AND Q20

Q19. How confident are you that when a coronavirus vaccine becomes available, it will have been properly tested for safety and effectiveness?

	12/20	09/20[13]
Very/Somewhat confident (NET)	70	55
Very confident	24	18
Somewhat confident	46	37
Not too/Not at all confident (NET)	29	43
Not too confident	18	26
Not at all confident	11	17
Don't know	1	2
Refused	*	1
Web Blank	-	*
	n=1,676	*n=1,769*

Q20. How confident are you that when a coronavirus vaccine becomes available, it will be distributed in a way that is fair?

	12/20	09/20[14]
Very/Somewhat confident (NET)	67	52
Very confident	22	16
Somewhat confident	45	36
Not too/Not at all confident (NET)	31	46
Not too confident	20	26
Not at all confident	11	20
Don't know	2	2
Refused	*	*
Web Blank	-	*
	n= 1,676	*n=1,769*

Q21. How confident are you that the development of a coronavirus vaccine is taking the needs of Black people into account?

Based on total Black adults

	12/20	09/20[15]
Very/Somewhat confident (NET)	49	33
Very confident	11	12
Somewhat confident	39	21
Not too/Not at all confident (NET)	48	65
Not too confident	23	34
Not at all confident	25	31
Don't know	2	1
Refused	1	1
Web Blank	-	*
	n=390	*n=777*

[13] Trend from KFF Undefeated Survey on Race and Health, September 2020

[14] Trend from KFF Undefeated Survey on Race and Health, September 2020

[15] Trend from KFF Undefeated Survey on Race and Health, September 2020

Q22. How confident are you that the development of a coronavirus vaccine is taking the needs of Hispanic or Latino people into account?

Based on total Hispanic adults

	12/20
Very/Somewhat confident (NET)	60
Very confident	16
Somewhat confident	44
Not too/Not at all confident (NET)	36
Not too confident	23
Not at all confident	13
Don’t know	3
Refused	1
Web Blank	-
	n=298

READ TO ALL: Now I have a few questions we will use to describe the people who took part in our survey...

AGE. What is your age?
AGE2. (ASK IF DON’T KNOW OR REFUSED AGE) Could you please tell me if you are between the ages of... (READ LIST)

RECAGE2 VARIABLE

	12/20
18-29	21
30-49	33
50-64	25
65+	21
Don’t know/Refused (NET)	1

CHILD. Are you the parent or guardian of any child under the age of 18 living in your household?

	12/20
Yes	30
No	69
Don’t know/Refused (NET)	1
Don’t know	-
Refused	1

MARITAL. Are you currently married, living with a partner, widowed, divorced, separated, or have you never been married?

	12/20
Married	43
Living with a partner	10
Widowed	7
Divorced	10
Separated	3
Never been married	26
Don’t know/Refused (NET)	1
Don’t know	*
Refused	1

RVOTE. Are you registered to vote at your present address, or not?

	12/20
Yes	80
No	19
Don't know	*
Refused	*

VOTED. Thinking about the presidential election that took place November 3rd, did you happen to vote in the election, including voting by mail, early voting, or absentee ballot before the election, or did things come up that kept you from voting?

Based on registered voters

	12/20
Yes, voted	91
No, did not vote	8
Don't know	*
Refused	*
	n=1,403

VOTED2. In the election for U.S. president, did you vote for (Donald Trump) or (Joe Biden), or someone else? (rotate text in parentheses)

Based on those who voted in 2020 presidential election

	12/20
Donald Trump	38
Joe Biden	51
Someone else	3
Don't know	1
Refused	8
	1,302

VOTED/VOTED2 Combo table

Based on registered voters

	12/20
Voted in presidential election	91
Donald Trump	34
Joe Biden	47
Someone else	2
Don't know/Refused	8
Did not vote in presidential election	8
Don't know/Refused	*
	n=1,403

INCLOSSTOTAL. Since February, have you or another adult in your household lost a job, been placed on furlough, or had your income or hours reduced because of the coronavirus outbreak, or not?

	12/20
Yes	46
No	53
Don't know/Refused (NET)	*
Don't know	*
Refused	*

EMPLOY. What best described your employment situation today?

	12/20
Employed (NET)	56
Employed full-time	43
Employed part-time	13
Unemployed (NET)	11
Unemployed and currently seeking employment	8
Unemployed and not seeking employment	3
A student	4
Retired	18
On disability and can't work	6
Or, a homemaker or stay at home parent?	5
Don't know/Refused (NET)	1
Don't know	1
Refused	*

ESSENTIAL. Have you been deemed an essential worker, meaning you are still required to work outside your home during the coronavirus outbreak?

Based on total employed

	12/20
Yes	66
No	33
Don't know/Refused (NET)	1
Don't know	1
Refused	-
	n=872

HCWORKER2. Do you or anyone in your household work in a health care delivery setting, such as a doctor's office, clinic, hospital, nursing home, or dentist's office?

	12/20
Yes (NET)	16
Yes, respondent	6
Yes, someone else in household	8
Yes, both respondent and someone else in household	1
No one in household	84
Don't know/Refused (NET)	1

HCWORKER3. Does (your/your family member's/you or your family member's) work involve direct contact with patients or their bodily fluids?

Based on those who say they or family member work in health care

	12/20
Yes	70
No	28
Don't know/Refused (NET)	2
Don't know	2
Refused	-
	n=255

HCWORKER2/HCWORKER3 Combo table

	12/20
Yes, respondent or family member works in health care delivery setting	16
Yes, respondent or family member's work involves direct contact with patients or their bodily fluids	11
No, respondent or family member's work does not involves direct contact with patients or their bodily fluids	4
Don't know/Refused	*
No, respondent or family member does not work in health care delivery setting	84
Don't know/Refused (NET)	1

COVERAGE. Are you, yourself, now covered by any form of health insurance or health plan or do you not have health insurance at this time? (READ IF NECESSARY: A health plan would include any private insurance plan through your employer or a plan that you purchased yourself, as well as a government program like Medicare or [Medicaid/Medi-CAL])?

	12/20
Covered by health insurance	86
Not covered by health insurance	13
Don't know	1
Refused	*

AGECOV VARIABLE

	12/20
Insured less than 65	83
Uninsured less than 65	17
	n = 1,165

COVTYPE. Which of the following is your MAIN source of health insurance coverage? Is it a plan through your employer, a plan through your spouse's employer, a plan you purchased yourself either from an insurance company or a state or federal marketplace, are you covered by Medicare or (Medicaid/[INSERT STATE-SPECIFIC MEDICAID NAME]), or do you get your health insurance from somewhere else? [INTERVIEWER NOTE: IF R SAYS THEY GOT INSURANCE THROUGH HEALTHCARE.GOV, OBAMACARE, OR A STATE HEALTH INSURANCE MARKETPLACE/EXCHANGE, CODE AS 3].

Based on those who are insured

	12/20
Plan through your employer	35
Plan through your spouse's employer	9
Plan you purchased yourself	9
Medicare	23
Medicaid	12
Somewhere else	6
Plan through your parents/mother/father (Vol.)	6
Don't know	1
Refused	*
	n=1,467

COVERAGE/COVTYPE Combo Table

Based on total

	12/20
Covered by health insurance	86
Employer	30
Spouse's employer	8
Self-purchased plan	7
Medicare	19
Medicaid	10
Somewhere else	5
Plan through parents/mother/father (Vol.)	5
Don't know/refused	1
Not covered by health insurance	13
Don't know/Refused	1

AGECOVTYPE VARIABLE

Based on those ages 18-64

	12/20
Covered by health insurance	82
Employer	36
Spouse's employer	9
Self-purchased plan	8
Medicare	6
Medicaid	12
Somewhere else	5
Plan through parents/mother/father (Vol.)	6
Don't know/refused	1
Not covered by health insurance	17
Don't know/Refused	1
	n=1,174

COVSELFOTHER. Regardless of your source of health insurance coverage, did you purchase your plan yourself?

Based on those who get health from somewhere else

Insufficient sample size to report.

Q23. Do you happen to know the deadline for individuals to sign-up for health insurance?

Based on those under the age of 65 and purchase their own insurance or uninsured

	12/20
Correct date	14
Other answer	12
Deadline already passed	1
There is no deadline	3
Don't know/Refused	70
	n=313

RSEX. Are you male or female?

	12/20
Male	49
Female	51
Other (Vol.)	*
Don't know	-
Refused	*

LGBT. Do you consider yourself to be gay, lesbian, bisexual, or transgender?

	12/20
Yes	7
No	91
Don't know	*
Refused	2

Q24. Do you normally get a flu vaccine each year, or not?

	12/20	09/20[16]
Yes	53	57
No	46	42
Don't know	*	*
Refused	*	*
Web Blank	-	*
	n=1,676	*n=1,769*

DELAYEDCARE. In the past 30 days, have you or a family member in your household skipped or postponed any type of medical or dental care due to the ongoing coronavirus outbreak, or not? IF YES ASK: And was that you, a family member, or both you and a family member who skipped or postponed medical or dental care?

	12/20	10/20
Yes (NET)	25	25
Yes, respondent	10	9
Yes, someone else in household	5	4
Yes, both respondent and someone else in household	9	12
No one in household has serious health condition	75	74
Don't know/Refused (NET)	*	1
Don't know	*	1
Refused	*	*
	n=1,676	*n=1,207*

TEST1. Do you personally know anyone who has tested positive for coronavirus, or not?

	12/20	Late 4/20
Yes	72	24
No	28	76
Know some whose test is pending (Vol.)	-	-
Don't know/Refused (NET)	*	*
Don't know	*	-
Refused	*	*
	n=1,676	*n=1,202*

Q25. Do you personally know anyone who has been hospitalized due to coronavirus, or not?

Based on those who know anyone who has tested positive

	12/20
Yes	54
No	46
Don't know	*
Refused	-
	n=1,168

[16] Trend from KFF Undefeated Survey on Race and Health, September 2020

TEST1/Q25 Combo table

	12/20
Yes, know someone who tested positive	72
Yes, know someone who has been hospitalized	39
No, do not know someone who has been hospitalized	33
Don't know/Refused	*
No, do not know some who tested positive	28
Know some whose test is pending (Vol.)	-
Don't know/Refused (NET)	*
Don't know	*
Refused	*

TEST3. Do you personally know anyone who has died from complications related to coronavirus, or not?

	12/20	9/20	6/20	Late 4/20
Yes	30	24	17	9
No	70	76	83	91
Don't know/Refused (NET)	*	1	*	*
Don't know	*	-	*	*
Refused	*	1	-	*
	n=1,676	*n=1,199*	*n=1,296*	*n=1,202*

Q26. Were any of the people you know who died from coronavirus family members or close friends, or not?

Based on those who personally know anyone who has died from coronavirus

	12/20	9/20
Yes	67	64
No	33	36
Don't know/Refused (NET)	1	-
	n=553	*n=290*

CHRONICCOVID. Do you or anyone in your household have a serious health condition such as high blood pressure, heart disease, lung disease, cancer or diabetes, or not?

	12/20
Yes (NET)	46
Yes, respondent	22
Yes, someone else in household	15
Yes, both respondent and someone else in household	10
No one in household has serious health condition	53
Don't know/Refused (NET)	1
Don't know	*
Refused	*

PARTY. In politics today, do you consider yourself a: (Republican), (Democrat), an Independent, or what? (rotate items in parentheses)

	12/20
Republican	25
Democrat	32
Independent	30
Or what/Other/None/No preference	8
Don't know	2
Refused	3

PARTYLEAN. Do you LEAN more towards the (Republican) Party or the (Democratic) Party? (rotate items in parentheses in same order as PARTY)

Based on those who are not Republican or Democrat

	12/20
Republican	29
Democratic	35
Independent/don't lean to either party (Vol.)	21
Other party (Vol.)	2
Don't know	7
Refused	6
	n=677

Summary PARTY and PARTYLEAN

Based on total

	12/20
Republican/Lean Republican	38
Democrat/Lean Democratic	47
Pure Independent	11
Undesignated	4

Five-Point Party ID

	12/20
Democrat	32
Independent Lean Democrat	15
Independent/Don't lean	9
Independent Lean Republican	13
Republican	25
Undesignated	6

IDEOLOGY. Would you say your views in most political matters are liberal, moderate, or conservative?

	12/20
Liberal	26
Moderate	36
Conservative	32
Don't know	4
Refused	2

EDUC. What is the highest level of school you have completed or the highest degree you have received? (DO NOT READ LIST) [INTERVIEWER NOTE: Enter code 3-HS graduate if R completed vocational, business, technical, or training courses after high school that did NOT count toward an associate degree from a college, community college or university (e.g., training for a certificate or an apprenticeship)]

	12/20
HS grad or less (NET)	38
Less than high school (Grades 1-8 or no formal schooling)	4
High school incomplete (Grades 9-11 or Grade 12 with no diploma)	4
High school graduate (Grade 12 with diploma or GED certificate)	30
Some college (NET)	30
Some college, no degree (includes some community college)	18
Two-year associate degree from a college or university	12
College grad+ (NET)	31
Four-year college or university degree/Bachelor's degree	18
Some postgraduate or professional schooling, no postgraduate degree	1
Postgraduate or professional degree, including master's, doctorate, medical or law degree	12
Don't know/Refused (NET)	1

INCOME. Last year – that is, in 2019 – what was your total family income from all sources, before taxes? Just stop me when I get to the right category. (READ LIST)

	12/20
Less than $20,000	15
$20,000 to less than $30,000	10
$30,000 to less than $40,000	11
$40,000 to less than $50,000	9
$50,000 to less than $75,000	12
$75,000 to less than $90,000	8
$90,000 to less than $100,000	6
$100,000 or more	22
Don't know/Refused (NET)	9

HHADULTS. How many adults, age 18 and over, currently live in your household including yourself?

	12/20
1	21
2	48
3	18
4	8
5	4
6 or greater	1
Don't know/Refused (NET)	1

KFF

Headquarters and Conference Center
185 Berry Street, Suite 2000
San Francisco, CA 94107
650-854-9400

Washington Offices and Conference Center
1330 G Street, NW Washington, DC 20005
202-347-5270

This publication is available at kff.org.

Filling the need for trusted information on national health issues, KFF (Kaiser Family Foundation) is a nonprofit organization based in San Francisco, California.
